# Book Reviews

**Published:** 1804-05-01

**Authors:** 


### CRITICAL ANALYSIS
OF THE
### RECENT PUBLICATIONS
ON THE
### DIFFERENT BRANCHES OF PHYSIC, SURGERY, AND MEDICAL PHILOSOPHY.

*Researches into the Properties of Spring Water, with Medical Cautions (illustrated by Cases) against the Use of Lead in the Construction of Pumps, Water Pipes, Cisterns, &c. by* WILLIAM LAMBE, M.D. *late Fellow of St. John's College, Cambridge.* London, pp. 204.

THE main object of the author in this Research is, to spread an alarm (if well founded, a very wholesome alarm) of the deleterious properties properties of all kinds of water in common use, when kept even in slight contact with lead. It may be considered as extending the cautions relating to this noxious metal so admirably laid down by Sir G. Baker, by Dr. Heberden, and some other sagacious physicians; but Dr. Lambe carries his enquiries to a much greater extent than has hitherto been done, and his cautions implicate the prudence of almost every individual domestic economy in the supply of that most important of all necessaries of life, water.

"Have we any reason to think," the author asks, "that this fluid can receive a contamination from the substances with which it is frequently brought into contact? Of all these substances Lead is that which is most commonly used, and is preferred to other metals, on account of its cheapness, the ease with which it fuses, and its great ductility, fitting it to be readily moulded into convenient forms. But these advantages may be esteemed rather to be a public calamity, if the metal communicate to the fluid its own deleterious properties. The question then, whether water in its ordinary condition has a power of dissolving lead, is, from the frequent use which is made of it, of the first importance to society.

"The general practice of the country seems to have decided this question in the negative. Whatever may be the suspicions of some, they have not been sufficiently strong to produce much effect on the public mind, nor scarcely any influence on our domestic arrangements. Many towns are principally supplied by water conveyed from a distance by leaden pipes; families preserve it in cisterns formed of lead; some public breweries, I am told, have similar cisterns on a large scale, in which the water is allowed to stagnate; pumps so formed are put down every day, in every situation, and in all sorts of wells; and I know not that any one asks the question, whether or not, by these arts, a large portion of the public is daily and hourly consuming a quantity of the most virulent and insidious poison."

The subject involves several important questions, and the author very properly considers the evidences of disease being produced solely by certain waters contaminated by lead, the diagnostic symptoms of this disease in different constitutions, and especially the proofs afforded by chemical examination of the existence of lead in these suspected situations.

The latter question furnishes the principal original matter in this volume. After giving a slight sketch of the *colica pictonum*, and the modifications of the poison of lead in different constitutions, the author observes, that it will naturally be asked, "If leaden machinery for conveying water is really so mischievous as is supposed, how happens it that the saturnine colic is not common over the whole kingdom, and endemial in those places, where this machinery is in common use? The objection is certainly so far well founded, that it must be acknowledged, that the saturnine colic, though probably much more frequent than is commonly supposed, is but of of rare occurrence, nor are even the pains of the bowels, which participate of its nature, by any means frequent, regard being had to the great number of persons, who are in the daily habit of using such waters.

" There are two reasons which may be given for this fact. The first is the minuteness of the dose. In my assay of a water, which I suspect to contain much more lead than is common, I calculate that the quantity dissolved is little more than a tenth of that which has been said to be contained in wine adulterated by litharge.

" A second obstacle, and one which is perhaps still more powerful, is the peculiar manner in which the metal is combined, as it is found in these solutions. This is not the place to enter at large upon this subject: it is enough at present to observe, that it is involved more or less completely in all of these waters with a matter, which seems to be of a vegetable nature. This union is in many cases so perfect, as to require the application of powerful chemical agents to dissolve the combination, and exhibit it under its common forms and relations. Thus it would appear, that the stomach and intestines are defended from its immediate action, in some cases totally, in all partially; and thus are the pains and spasms, which are the consequences of this immediate action, prevented."

Pursuing this idea of the very gradual introduction of this poison, he observes on the permanence of the symptoms, " the diseases engendered by lead seem to be in their nature lingering and chronical. In cases where preparations of this metal have been given as a medicine, and its use continued but a very short time, it has been known to occasion a disease of such obstinacy, as to require a very considerable length of time for its removal. Thus, we are informed, that four grains of cerussa acetata, taken for no longer a time than three successive days, " occasioned intolerable uneasiness in the bowels for some months." It is a commonly received opinion, founded, I believe, on actual observation, that the longer the force of this poison has been accumulating before producing evident disease, the more dangerous has been the disease when produced, and the longer has been the time required for its effects to wear off. This is known to be the law observed, when it has occasioned saturnine colic, and in this instance we may safely confide in analogical reasoning, though the symptoms be anomalous. As in disease from poisoned waters, the evident mischief is much more slow and gradual than in all others, that have been hitherto noticed, the same law may be expected to hold good in a still higher degree. Accordingly, in the instances which have occurred to myself, the restoration to health has been very gradual: three or four months have been required, before a considerable amendment has taken place; a twelvemonth or more, before the complete re-establishment of the health."

Several cases are given of obstinate and in a few instances fatal diseases, which the author ascribes to this metal; some of these cases do not appear altogether satisfactory. The medical part concludes cludes with the following recapitulation: "Such is the result of my own experience, and of a few authentic facts which have come within my knowledge. I think it established by these observations, that the solutions of lead by spring water are sufficiently powerful to excite every one of those symptoms, which are allowed, by the first medical authorities, to be the genuine and specific offspring of this poison. These are the peculiar and excessive pains of the stomach and intestines, severe muscular pains principally seated in the fore arms, and the paralytic weakness of the limbs, particularly of the hands and arms. Besides these, some other forms of disease have, I hope, been fully and satisfactorily traced to the same exciting cause. Some of these must as yet be deemed anomalous, as not being received into the established classes of systematic writers.

"I cannot but conclude, that lead ought never to be brought into permanent contact with water, which is intended in any form to be received into the human body.

"I might adduce very many other instances of disease, which I believe either to have originated, or to have been greatly aggravated by the same cause. I am particularly convinced that it gives a strong disposition to apoplexy. Many paralytic tremors, obstinate constipations, perpetual vomitings, tympanies, tumors of the abdomen in females, glandular swellings and schirrosities, have I believed to have been so caused, exasperated, or rendered fatal."

The author concludes with giving the chemical experiments which have enabled him, as he supposes, to detect lead in minuter proportions than any hitherto known method has been able to do.

The common test for this metal is sulphuret of potash, or water impregnated with sulphurated hydrogen gas. These tests, added to an extremely weak solution of lead, turn it first brown, then black, and by repose a dark sediment subsides. The author asserts, however, that where the quantity of lead is too small to be indicated by the simple application of the test of sulphurated hydrogen, it may be detected in the manner described in the following passage: "In operating on these waters, I have distinctly noticed four different appearances.

"(A) The test forms a dark cloud with the precipitate, re-dissolved in nitric acid.

"(B) Though it forms no cloud, the precipitate itself becomes darkened by the test.

"(C) The test forms a white cloud, with the precipitate treated as in (A). These two last appearances may be united.

"(D) The test neither forms a cloud, nor darkens the precipitate.

"(E) In the cases (B, C, D,) heat the precipitate, in contact with an alkaline carbonate, to redness, dissolve out the carbonate by water, and treat the precipitate as in (A); then the test forms a dark cloud with the solution of the precipitate. In these experiments it is necessary that the acid used to re-dissolve the precipitate be not in excess; if it should so happen, the excess must be saturated, before before the test is applied. It is better to use so little, that some precipitate may remain undissolved."

Another way of using the test, which the author proposes, is to add muriate of soda to precipitate the lead in the form of a muriate, to heat this to redness with carbonate of soda, dissolve out the superfluous alkali, treat the residue first with nitric acid, and lastly with the sulphureous test.

A third method is by reduction of the metal, and certainly the most satisfactory, but inconvenient from the great bulk of water to be analysed in order to obtain sufficient lead to decide its identity.

With regard to the time necessary for giving spring water a sensible saturnine impregnation, when in contact with lead, the author thinks that a very few hours are sufficient, to judge by experiment.

Some other chemical observations are added, for which we must refer the reader to the work itself; we are compelled to add, however, that some very unsatisfactory inferences are given, and many of the author's experiments are too unfinished, and performed on too small quantities, to appear with that weight which the medical part of the volume required, and which perhaps their intrinsic value deserved.

The author has succeeded in making out a strong case for the public attention; and the specimen before us leads us to hope that he will consider himself engaged to pursue a subject which applies perhaps as extensively as any other single cause of disease, and in a form the most likely to elude suspicion.

---

*Discourses on the Management of Infants, and the Treatment of their Diseases, written in a plain familiar Style, to render it intelligible and useful to all Mothers, and those who have the Management of Infants. By* John Herdman, M. D. Edinburgh.

The first article in the sum total of every yearly bill of mortality must strike the most superficial observer with astonishment mixed with a degree of horror, *more than one fourth of the human race perish in the first year of life!* To what is this waste of human life to be ascribed? Is it a necessary part of the general plan of human reproduction? or is it owing to culpable negligence, mistaken notions, or ill directed care in the parents? The author in this popular address to mothers entirely adopts the latter opinion.

"Is it appointed, in the nature of things, that so great a proportion of mankind are born to die in infancy? No, the idea is impious, and directly arraigns the very Author of Being. He has formed them to live, and their premature death is none of his doing. It is owing to mistaken notions, and misguided reason; to a miserable and a total oversight of all the institutions and intentions of Nature, in regard to their treatment; to practices the most absurd and unfounded; in one word, to the most horrid and culpable mismanagement."

The author here speaks the truth, but not all the truth; that which which he adduces as the cause of all this mortality, is only one of its causes, extensive indeed, but by no means so prominent as to be thus exclusively mentioned. Hereditary disease, or at least tendency to disease, defective stamina, absolute neglect and want of natural tenderness on the part of the parents, and many other causes conspire to produce this formidable sum total of destruction. However we shall not dwell any longer on this question, for the reader must be well aware that these are causes which no popular discourse like the present can ever reach; and the precise evil which the author opposes, is sufficiently extensive to excuse this partial exaggeration, if it can increase the force of the appeal. To those mothers therefore who eagerly wish to bestow every care upon their helpless offspring, even to those who are much more likely to *kill with kindness* (to use a popular phrase) than to injure by indifference and neglect, this appeal is addressed; and the importance of the subject gives an interest and dignity even to the minutest detail of the nursery.

The author begins his plans of reform by an attack on the matrons of the lying-in chamber.

"There is one thing in which you err, and that is, your implicit confidence in the judgment and opinions of your midwives and nurses. You allow and desire them to prescribe, not only for your infants, but also for yourselves. You take them out of their own sphere, and you dignify them with the office of the Physician. They are employed about they know not what, and they deal destruction around them.

"On the other hand, if you are willing to give them consequence, they are as willing to take it. They consider the management of you, and your infants, as their own peculiar province; they are jealous and fearful of the least interference; they give a profusion of advice, but they take none; they insinuate that it is they, and they only, that know any thing of the matter; they are wise in their own conceit, and their conceit and obstinacy are only equalled by their ignorance."

Having enlarged upon the incapacity and bigotted folly of the matronly counsellors, we are introduced to one whom the author looks up to with implicit confidence.

"But I must now tell you, that the fit and proper management of the infant state, is not to be found in experience alone. It is to be found in more sure and infallible grounds, than either experience or reason; it is to be found in the fixed and established laws of Nature; in laws which Nature has implanted in the breast of every living being, human or brute, and which are no less stable than the very foundations of the earth itself: in one word, the fit and proper management of the infant state, is to be found in the sure and unerring principle of *instinct.*"

This has often been said, and in support of the all-sufficiency of instinct the example of inferior animals has constantly been adduced, whence the following inference:

 "Thus

"Thus, you may learn, that, in the general œconomy of life, reason is a frail and erring guide, and that instinct is a much more sure and certain director. Observe also, that those animals more immediately under the direction of man, as horses, cows, and other tamed animals, are much more subject to disease and death than wild animals, or the same species in the wild state. When left to the direction of their natural instincts, they enjoy uninterrupted health; but, subjugated to the caprice of man, they become partakers of his calamities."

However, it will always be answered to such general reasoning, that the customs of the society in which we live (whether right or wrong) have given rise to circumstances totally artificial, to which mere instinct can never apply: the domestic animals subservient to the use of mankind are themselves artificial; and it is only by thwarting many of their natural instincts that we can make them what we want.

But to proceed to particulars; for it is easy to say to parents, "your infants are subject to fatal diseases in exact proportion to the progress of your luxuries and supposed refinements;" the truly valuable secret of rearing children is to ensure vigorous health in the midst of these luxuries and refinements; and since, in such a state of society, the dictates of instinct will not suffice even for the management of the nursery, reason and experience must be called in to supply the deficiency.

The first custom which the author declaims against, is that of washing new-born children. The objection is at least original, we shall therefore give it in the author's words.

"The infant born, the first thing that engages the attention of the good woman in attendance, the midwife, or nurse, is the cleansing of his skin. This is reckoned a most necessary piece of duty, and much does he suffer by the operation.

"He suffers from no less than five causes, First, from exposure to cold. Secondly, from being tossed and tumbled about upon the nurse's knee. Thirdly, by friction by her rough and rude hands. Fourthly, from the nature of the cleansing substance. And fifthly, he suffers, and he suffers most severely, from the excoriations and inflammations which follow this officious cleansing of the skin.

"But, why should all these things be done? Why should the infant's skin be washed or cleansed with spirits, or wine, or ale, or butter, or pomatum, or soap and water, or plain water, or any other substance whatever? All these, and more, have been, at one time or another employed; yet, why should any one of them be employed? or, why should the infant's tender and delicate skin be subjected to such treatment? If there be any good reason why it should be done, let it be done; but, if there be not, let it be left undone.

"This coating or covering which the infant obtains in the womb is surely not put there for nothing. Be assured that Nature has some wise and necessary design or purpose in the matter; for it adheres, heres, and it adheres most firmly to his skin: and, if left to itself, in a certain period after birth, it dries, and forms a crust, and gradually scales off in the œconomy of Nature, and leaves the skin it covered, heal and healthful, and capable to bear every common or necessary freedom.

"The parts of the infant's body most subject to excoriation and inflammation are the parts which come in contact with each other, where skin is applied to skin, as the neck, arm-pits, and groins. Now, on these parts, the natural coating, the covering which the infant obtains in the womb, is thicker than on almost any part else. Nature, conscious as it were of excoriation and inflammation in the neck, arm-pits, and groins, has provided against these ills, yet are her intentions frustrated by ignorant and officious care.

"But, suppose, if you please, that this covering of the infant's skin is intended for no purpose, suppose that it neither serves the purpose of warmth, nor defence, nor prevents fretting nor inflammation, yet why should it be removed? It is neither poisonous, pestilential, nor deadly; it will neither contaminate bed-clothes nor body-clothes, nor will it cause disease; it is as innocent as the infant it covers. As I have already told you, it dries, and forms a crust, and gradually scales off in the œconomy of Nature, and leaves the infant's skin heal and healthful, and capable to bear every common or necessary freedom.

"Disease, however, is a necessary consequence of its removal. It adheres, and it adheres most firmly to the proper skin. It is as it were a first skin, to be thrown off, in a certain period after birth, when the proper skin becomes fitted to perform its office, or to bear the action and contact of external things. It cannot be touched without injury. Nay, it adheres so firmly to the head, the neck, the arm-pits, and groins, that it cannot be removed without removing the skin itself. But the skin is injured by its removal in any degree; it is injured by the rubbing of the hand, and by the substance employed in its removal. These causes bring excoriations and inflammations and severe pain. Then comes the need of your drying and absorbing powders, your white lead, your tutty, and your burnt rags. By this officious cleansing of his skin, your midwives and nurses bring much suffering to the infant, and much trouble to themselves.

"What, then, have they to do? They have nothing to do but to take the infant's skin as Nature gives it them; nothing to do but to dry it, in the most kind and gentle manner, with the receiver, or a piece of old soft spongy cloth, warmed at the fire, and then proceed to clothe him."

Whether cleanliness be a natural instinct or an acquired habit, as it forms one of the best refinements and greatest decencies of civilized life, we should be sorry to see an iota of it given up to such an idle hypothesis. Let the author, if he pleases, exclaim against wine, ale, brandy, and chilling lotions; they are to be condemned, chiefly because they do not answer the purpose of cleanliness so well as simple warm soap and water; and we are certain that there is not a decent lying-in chamber in the kingdom, in which such systematic want of neatness would not be heard with utter disgust and abhorrence. We fully believe that bodily purification, of herself and her offspring, is an universal *instinct* with the female sex, under such circumstances; and this extends to inferior animals, as will occur to every one who is acquainted with the process of birth, without our enlarging on a subject which, on no other occasion, we should have thought it seemly to introduce. With regard to the imagined injury done to the infant's skin, one would think, by the author's representation of it, that the poor child had undergone the operation of sand and a scrubbing-brush, and not a gentle friction with the nurse's soaped hand; which, rough as it may be, is gentler, and less excoriating than the finest damask towel.

Clothing is the next circumstance that engages the Author's attention, and calls forth his censures. Here, however, he is aware that he is not treading a new path; most of the absurdities and miserable consequences of the old system of swaddling and tight bandaging *have been* corrected, and all that is urged from the example of uncivilized nations for the benefit of ease and unconfined clothing, would have applied with much more propriety a century ago than to the present day. Beyond this single point the comparison will not hold; the Negroes, the Caffres, the Egyptians, whom the Author cites with so much approbation, have a very easy task to rear their children in a climate where all clothing is unnecessary, and is actually never put on till the age of puberty; where the new-born infant may bask naked in the sun, and sprawl about at pleasure on the warm sand. But what has this to do with the arduous task of nursing the child of the bleak and foggy North? the offspring of civilized society inheriting a delicate *town-bred* constitution, and from necessity immured in the confined streets of a populous city.

The following simple clothing is advised.

"On these principles, therefore, let the new-born infant be clothed in the following plain, simple, and easy manner. Let his head be covered with two caps, the first of flannel, and the second of cotton; and let the first be slightly stitched within the second, forming a lining to it as it were. Thus they may be both put on at once, and the uppermost may be tied loosely below the infant's chin. Then let his whole body, as well as his arms and legs, be covered with the finest cotton cloth, by way of shirt, made loose and easy, and to tie before; and then let him be wrapped in a fine, soft, and warm flannel wrapper, and laid to rest; laid into your own bosom, or into the bosom of a nurse."

In the usual dress of an infant a bandage is put round the belly, chiefly with a view of supporting the navel, and preventing umbilical protrusion. We are disposed to agree with the Author, that the idea of danger of rupture during the separation of the small portion portion of umbilical cord that is left, is entirely without foundation.

The feeding of the infant next engages the Author's attention. He mentions, with just reprobation, the many absurd ideas, and still more absurd practices, relative to the expulsion of the meconium by purgatives. Our readers need not be reminded, that the first stools of the healthy infant contain this thick brown matter called *meconium*; and that the first milk afforded by the mother has a gently purgative quality, which favours the expulsion of this peculiar matter from the infant's bowels.

The long retention of the meconium is generally allowed to disorder the infant, and hence the utility of early purgatives in those cases only where the child is debarred of its natural food. But the Author very properly objects to the common practice of cramming children as soon as born, and intended to suck their mother, alternately with unnatural physic and unnatural food, when the first element that Nature has provided for them is at the same time wholesome food and salutary physic. We know it is a mighty difficult task to persuade the good women, that a new-born child does not come into the world hungry, and the sole interpretation that they can give to its cries is food! food! food! Though the peculiarities of the fœtal circulation would not be so well understood in the nursery, every sensible mother must at least feel the force of the following appeal.

"Such are the effects of the artificial purgation of the infant; and such also are the effects of another practice to which he is subjected; I mean, the feeding him with wheys, panadas, and gruels, before he is applied to the breast.

"The source of your error is this. You think that the infant may starve before you are ready to give him the breast. But this is a vain thought, for he is in no danger of starvation; his blood is rich in nourishment, as it were; he had a constant supply till the moment of his birth; and this supply is fully sufficient for the purposes of his œconomy till the changes of his birth are effected; till his own organs, his stomach and bowels, are ready to digest and prepare his nourishment, and till you are ready to give him the breast. Besides, how can you suppose that Nature would leave a matter, so essential to the welfare, the preservation, and existence of the infant, so ill contrived? And pray what do the young of the inferior animals get before the mother's milk is ready for them? No one thing whatever; it is ready so soon as they require it, or so soon as they are ready to receive it.

"This is a universal law of Nature, a law which obtains with you, as well as with the inferior animals, that the milk of the mother is in preparation, and in readiness, at the very time her offspring require it, or are ready to receive it; or, in other words, the infant's necessity for food, and the mother's ability to supply it, exactly keep pace or correspond with each other."

The next subject is one in which the feelings of mothers are peculiarly culiarly engaged, a subject which has often employed the pen both of the most eloquent and of the most experienced writers; it is, the duty and propriety of mothers giving suck to their own offspring. The very possibility that such a question should ever have been agitated, implies some great, some radical defect in the present state of society. The Author scarcely assumes the tone of the moralist, but merely that of the medical adviser; his object is not so much to persuade the wavering mother that she *ought* to devote herself to her child, as to convince her that she can do it fafely, however delicate her own constitution may be, and that the health of her infant imperiously demands it. He thus addresses her.

"What, then, can prevent you from nursing, when you have not been mismanaged; when you fall into no disease; when the secretion of your milk takes place in the natural and ordinary course? Yet, even in this case, you frequently say that you have not a sufficiency of milk! or that it is so poor and thin, and not sufficiently nourishing!! or windy and unwholsome, causing disorder and disease in the stomach and bowels of the infant!!! On one or other of these grounds you either do not nurse at all, or you nurse, but feed the infant also with other substances; with panadas, gruels, &c.

"But let me ask, are you not a mother? Have you not nursed and nourished your infant in the womb? And have you not brought him into the world naturally and properly formed; a mature and a perfect infant? You have performed the one half and the first part of your duty, and you have performed it naturally and properly. Why, therefore, should you not perform the last part as well as the first? Are your fluids more scanty after the infant's birth than they were before it; or more poor, or more thin, or less nourishing, or less wholesome? In a word, have you suffered any change since the infant's birth; or have you fallen into any disease? If not, why should you not nurse and nourish your infant at the breast as well as in the womb? There is surely no one thing to prevent it, unless there be something wrong in the original formation of your breasts, or unless you want nipples.

"Even the milk of another woman is not a proper food for your infant; for it is foreign to his nature; it is not a product of the fluids by which he was formed and nourished in the womb. Some time has also elapsed since another woman's delivery. Her milk is not the milk of a woman newly delivered; it has undergone a change; it is not adapted to the digestive powers of the new-born infant; it is considerably indigestible in his stomach; it produces diseases in his stomach and bowels, and which disease continues till by habit his stomach acquires the power of digesting it; a power, however, which it may not acquire during the greater part of the period in which he is suckled."

From a popular appeal, like the present, general arguments and general applications can only be expected; otherwise, we might observe that however advantageous it might be to the health of the most most delicate mother to suckle her infant, it does not necessarily follow that the child would receive equal benefit. When the author speaks of the nourishment within the womb, he seems to take it for granted that all children come into the world healthy and vigorous; but is it not fair to suppose that the slim, puny infant has *not* been duly nourished before birth? and that the first and most essential step to bring it into a thriving state is to substitute for its mother's, the nutritious fluids of a more vigorous nurse? In this we think we may confidently appeal to experience.

The management of temperature and exercise in the earliest months of infancy, and the time and mode of weaning, occupy the remainder of this little treatise. On the subject of exercise the following caution is given, which merits attention, as it often arises from anxious but mistaken care in the most tender mothers. It is the injurious practice of sending out very young infants in bleak ungenial weather, perhaps in intervals snatched between showers, to take the air in the arms of the nursery maid.

" But to send an infant abroad in the view of exercise is truly ridiculous; for where is the exercise of being carried motionless in a woman's arms? The exercise is to her, and not to the infant. By the exertion of walking, and of carrying the infant, the heat of her body is preserved, her feelings are kept agreeable, and she receives no injury; while the poor helpless innocent, motionless in her arms, is losing heat every moment; is starving alive as it were; is suffering all the pains and injurious effects of cold.

" Now, the difference between the woman who carries the infant, and the infant himself, must strike you. She can walk and exert her own muscles. By this power of action all the injurious effects of temperature are prevented, and she receives all the advantages and all the benefits of air and exercise. But the infant has no such power, and therefore cannot receive the true benefits of air and exercise till he acquires it; till he is able to walk and to exert his own muscles.

There is much good sense in this observation; it must strike every attentive observer who is constantly seeing the blue-cold, shivering, crying infant, paraded round the windy squares and open walks of this metropolis, in weather in which the grown up man quickens his pace and buttons up his great coat.

On the whole, we have perused this little treatise with pleasure and satisfaction; nothing indeed of importance is contained which is not found in Cadogan, Armstrong, and other guardians of infancy; the minutiæ of useful advice are almost entirely omitted, but the style is argumentative and the manner popular; and the mother, or future mother, that casts her eyes upon it, will not readily leave the perusal unfinished.

---

*History of the Proceedings of the Committee appointed by the General Meeting of Apothecaries, Chemists, and Druggists in London, for the Purpose of obtaining Relief from the Hardships imposed on the Dealers in Medicine, by certain Clauses and Provisions contained in the New Medicine Act, passed June* 3, 1802, *together with a View of the Act, as it now stands, in its ameliorated State; to which are added, the Substance of every Clause in the Acts of June* 3, 1802, *and July* 4, 1803, *and the Clauses of both these Acts, collated with each other, consolidated and explained; also a copious and carefully arranged Schedule. With explanatory Notes and Observations, by* William Chamberlaine, *Surgeon, Chairman of the Committee.* London.

The observations of which this pamphlet is composed, have already appeared in different Numbers of the Tenth Volume of this Journal. Mr. C. has here collected them, and made the most important addition of the Schedule, in which the list of the taxable articles is given. Of this Mr. C. thus speaks:

"And as the Schedule is part of the Act itself, so, conceiving that the work would, in some measure, be incomplete without it, I have, in a copious and carefully arranged Schedule, faithfully enumerated every article inserted in the Schedules of both Acts of Parliament, wherein are clearly and distinctly shewn all the articles in each, now liable to the medicine duty; all those which were made liable by the former Act of 1802, but are now exempt; and those which, though omitted in the Schedule of 1803, may, under certain circumstances, be liable to the tax.

"This, it is conceived, may not only afford material information to Druggists; Apothecaries, who keep shops for retail practice; country dealers, many of whom embark largely in the drug trade, and also merchants who export drugs to foreign parts, but may also be found of some use to Magistrates, before whom informations may be brought under the Medicine Act, as pointing out, as far as my judgment enables me, every circumstance wherein conviction should or should not follow. If, as we are all liable to error, (and especially, in cases where a writer, ignorant of law, presumes to discuss a subject connected with the law of the land), any errors may be found, all such as may be pointed out, shall be thankfully acknowledged and carefully corrected in a future edition, provided the demand for the present work should shew it to be of sufficient importance to the public to make a future edition necessary."

The history of these proceedings exhibits great sagacity, zeal, and good sense in the Committee, and on the part of Government much polite attention and liberal concession. Every person concerned in the sale of medicines in general must acknowledge the equitable spirit which actuated the latter, and should feel highly thankful to the Committee for having relieved him from the

2 harrassing harrasing uncertainty of the former law, and the unintentional oppression to which he was exposed in its execution.

To Mr. C. in particular, as a very active Chairman of the Committee, these thanks are especially due, and we doubt not will be generally felt, and willingly paid.

The Schedule is also curious, as it gives an authentic list of the most popular *shop* medicines in the kingdom, or those which are usually taken without the previous advice of a regular medical man.

---

*An improved Method of treating Strictures in the Urethra; by* THOMAS WHATELY, *Member of the Royal College of Surgeons, in London.* 8vo. pp. 230. London, 1804.

WE have on a former occasion (No. 27, p. 479, &c. and No. 28, p. 572, &c.) commended the zeal and industry of Mr. W. in improving the treatment of diseases of the urethra, and particularly his attention to safe and lenient means, in preference to the harsh, painful, and dangerous modes proposed by others. In the present work, which may be considered as a distinct treatise from the former on the same subject, the mildness and safety of the practice are carried much farther; probably, as far as they can be carried without becoming ineffectual or too tedious.

Mr. W. thus explains his own views. "The main design of the present work, is to recommend an improved method of treating strictures of the urethra. The public is already in possession of some remarks I made on Mr. Home's practice: but having seen, since their publication, additional objections to his method of treatment; the reader will find some further observations on it in the volume now before him. I should apprize him, however, that the animadversions contained in this treatise, relate entirely to what is advanced in his former volume on the subject: his second volume I did not see till these sheets were in the press. I observe that in this last work he has not attempted to refute any of the observations made upon his practice in my former essay; nor does he seem to pay much regard to the dreadful consequences so frequently produced by his mode of practice; but proceeds, without any material deviation from his former plan, by forcing a passage with a large armed bougie into the bladder. These consequences, in my opinion so pregnant with evil, are detailed, as in his former volume, with an indifference which might lead to the supposition that they are the unavoidable effects of the most judicious mode of treatment. I have differed from him in sentiment, and an imperious sense of duty to the public compels me to avow it. I have discharged that duty; and "I am very well satisfied," to adopt the language of Mr. Home, "that the public should judge between us by an impartial consideration of our works."

The first chapter is devoted to a general view of the subject; the different degrees or kinds of stricture; the usual causes, symptoms, and and consequences of stricture, and a description of the proper bougies.

The second chapter contains the author's new improvement, which we shall give in his own words. "In the preceding pages I have endeavoured to show, that strictures of the urethra are not merely contracted fibres, but really diseased portions of the membrane lining that canal, with a continued disposition to increased contraction. In this view of the disease, it seems probable, that the application of a remedy, calculated both to remove the diseased affection, and to dilate the contracted part, might perfectly cure the complaint, without putting the patient to the inconvenience of wearing a bougie. Such a remedy is caustic, when judiciously used. Hitherto the lunar caustic has been chiefly employed for this purpose, and my former work upon this subject will show the high opinion I entertain of this remedy, in the various diseases in which it is employed. It has, however, been my good fortune, to discover a more efficacious, and, at the same time, a less painful and hazardous remedy for the disease in question. This valuable remedy is the kali purum, which, if used in the manner, and with the precautions shortly to be described, will be found of singular efficacy in removing the complaint. I have already had so much experience of it, and am so perfectly convinced of it's superiority over the lunar caustic, as well as over the common bougie, that I now use it in a considerable number of the cases that come under my care. Of it's safety I am as well convinced as of it's efficacy; for, if used with circumspection, experience shows there is little danger of it's producing any disagreeable effect.

"Before a caustic of any kind is applied to a diseased surface, care should be taken, that the state of the parts, and the habit of the patient, are such as to warrant it's application. In almost every instance in which it is applied with these precautions, the benefit expected to be derived from it will accrue, without any risk whatever, and with much less pain than might be expected from a remedy of this nature. But if, on the contrary, these precautions be not taken; if the caustic be applied while the parts are in a highly inflamed or irritable state, or tending to gangrene; if the habit be bad, and the patient very far advanced in years; we may expect the most mischievous effects from its application. Under such circumstances, the use of any kind of caustic, in strictures of the urethra, is dangerous in the extreme. By a little attention, however, it will be a very easy matter to distinguish the cases fit for the use of this remedy, from those which are not.

"If the patient be affected with fever, or any other acute disease; if he be much indisposed, indeed, from any cause; if, in particular, he have a gonorrhœa, attended with much inflammation and irritation in the urethra; if the prepuce, glans, or any part of the penis, or the parts adjoining to it be swelled and inflamed; if the urethra, and especially if the strictured part of it, be so irritable as not to bear the touch of a bougie, the use of caustic is, for the present, forbidden.

forbidden. Great caution should likewise be observed in applying this remedy to those who are far advanced in years. There are, indeed, many elderly persons, who bear this remedy well; yet good reasons might be assigned, why age alone should make us cautious in applying caustic. But supposing none of these objections to exist, the application of caustic should not be the first act of the practitioner, on discovering a stricture in the urethra; some previous steps should be taken, in order to insure the benefit which it is capable of affording, and that without giving unnecessary pain, or producing the serious consequences which frequently follow it's injudicious application."

Mr. W. next explains the "previous steps," which he does at considerable length, and in which he shews great skill, address, and humanity.

He then proceeds with his improved method: "Having shown that this kali purum caustic ought not to be applied to strictures of the urethra, till a bougie of a proper size can be passed into the bladder; having likewise pointed out the methods to be taken previous to it's application in different cases of stricture; and enumerated certain cases and circumstances, under which this valuable remedy is interdicted, I proceed to state that mode of removing the complaint in question, which it is the particular object of this treatise to recommend. Our first business is to instruct the young practitioner how to arm a bougie with this caustic. For this purpose, put a small quantity of kali purum upon a piece of strong paper, and break it with a hammer into small pieces, about the size of large and small pin's heads. In doing this, care should be taken not to reduce it to powder. Thus broken, it should be kept for use in a vial, closed with a ground stopper. The bougie should have a proper degree of curvature given to it, by drawing it several times between the finger and thumb of the left hand.

"Before the caustic is inserted into it, it will be necessary to ascertain the exact distance of the stricture, to which the caustic is to be applied, from the extremity of the penis. For this purpose, let the bougie (which should be just large enough to enter the stricture with some degree of tightness) be passed, in a gentle manner, into the urethra; and when the point of it stops at the stricture, which it almost always does before it will enter it, make a notch with the finger nail, on the upper or curved portion of the bougie without the urethra, exactly half an inch from the extremity of the penis. When the bougie is withdrawn, a small hole, about the sixteenth part of an inch deep, should be made at the extremity of it's rounded end. A large blanket pin, two inches and a half in length, with the head struck off, will answer the purpose; the hole being made with the point of the pin. The extremity of the bougie should then be made perfectly smooth with the finger and thumb, taking care that in doing this the hole in its centre be not closed. Some of the broken caustic should then then be put upon a piece of writing paper, and a piece less than half the size of the smallest pin's head should be selected; the particle, indeed, cannot be too small for the first application. Let this be inserted into the hole of the bougie with a pocket knife, spatula, or some such instrument; and pushed down into it with the blunt end of the pin, so as to sink the caustic a very little below the margin of the hole. To prevent the kali from coming out, the hole should then be contracted a little with the finger, and the remaining vacancy in it be filled up with hog's lard. This last substance will prevent the caustic from acting on the sound part of the urethra, as the bougie passes to the stricture. Let the bougie be oiled, when it is completely prepared for the office it has to perform. Then let the operator, without delay, pass it by a very gentle motion, with the curvature upwards, to the anterior part of the stricture, upon which the caustic is to be applied. In doing this, the end of the bougie, that is held by the finger and thumb, should be a good deal inclined towards the abdomen, on its first introduction into the urethra, in order to preserve the curvature of the bougie. After it has passed about five inches, this end should be gradually brought downwards, as the bougie passes on, till it forms a right angle with the body. It will be known when the bougie arrives at the stricture, by the resistance to it's progress felt by the hand of the operator.

( To be continued. )

